# Diuretic drug utilization in neonates: a French prescription database analysis

**DOI:** 10.3389/fphar.2024.1358761

**Published:** 2024-03-13

**Authors:** Silvia Iacobelli, Simon Lorrain, Ezira Rabe, Béatrice Gouyon, Jean-Bernard Gouyon, Francesco Bonsante

**Affiliations:** ^1^ Néonatologie, Réanimation Néonatale et Pédiatrique, CHU Saint Pierre, Saint Pierre, France; ^2^ Centre d’Etudes Périnatales de l’Océan Indien (UR 7388), Université de La Réunion, Saint Pierre, France

**Keywords:** neonates, diuretics, pharmacoepidemiology, NICU, CPOE, furosemide, VLBW infants, fluid overload

## Abstract

**Background:** The use of diuretics is extremely common in infants cared for in neonatal wards, despite the lack of proven efficacy for many conditions. The main objective of this study was to assess the rate of diuretics exposure in a multicenter French cohort. The secondary objectives were to describe the evolution of this exposure over time, the indications, the prescription practices, and the exposure rates among centers.

**Methods:** An observational study was conducted in 40 Level 3 French neonatal intensive care units using the same computerized order-entry system. Neonates hospitalized between January 2017 to December 2021 with a corrected age between 24 and 44 weeks of gestation at admission were eligible.

**Results:** A total of 86,032 patients were included. The exposure rate was 8.5%, more specifically 29.4% for children born at < 32 weeks of gestation and 3.7% for neonates born at term. There was no significant variation over the study period, but the exposure ranged from 2.4% to 26.5% depending on the center. The main drugs prescribed were furosemide, spironolactone and dopamine with a diuretic purpose. The main indications were “fluid retention,” and to a lesser extent “bronchopulmonary dysplasia” and “post-transfusion.” For furosemide, the first exposure occurred in mean at 16.5 (±17.8) days of life, mean duration of exposure was 6.2 (±9.5) days, and the cumulative dose was in mean 10.7 (23.9) mg/kg.

**Conclusion:** Diuretic prescription practices vary between centers. The administration of these drugs is often non-evidence based, doses and duration of treatment easily exceed toxic thresholds.

## 1 Introduction

Diuretics are therapeutic agents that promote the excretion of water and electrolytes by acting on various sites along the nephron. These medications have several indications such as edematous disorders, arterial hypertension, and oliguric renal failure. The primary indication for diuretics in neonatology is water and sodium overload (Guignard and Iacobelli, 2021; Iacobelli, 2023). However, diuretics are commonly prescribed in neonatology units for pathophysiological conditions that are not necessarily associated with water and salt retention: transient tachypnoea of the newborn at term, hyaline membrane disease, hypercapnia, patent ductus arteriosus and bronchopulmonary dysplasia (BPD) (Segar, 2012). A national US web-based survey conducted with 400 neonatologists caring for very low birth weight infants, described the therapeutic strategies chosen for management in different clinical scenarios. This study reported diuretic use in 31% of scenarios, and concluded that among neonatologists, the “expectation” regarding these medications far exceeds the evidence-based data of their effectiveness (Hagadorn et al., 2011). Diuretic agents can be classified according to their mechanism of action into 7 groups: osmotic diuretics, loop diuretics, thiazides, carbonic anhydrase inhibitors, K^+^ -sparing diuretics, xanthines, and filtration diuretics (Guignard and Iacobelli, 2021; Iacobelli, 2023). Recently, an educational review on the use of diuretics in the neonatal period assessed more than 50 articles, including 7 meta-analyses, 2 systematic reviews and 6 clinical trials or prospective studies (Guignard and Iacobelli, 2021). This review indicated, for each class of diuretics, both therapeutic indications validated in the literature and clinical situations in which the use of diuretics is debatable or questionable (Table 1) (Guignard and Iacobelli, 2021; High Authority of Health, 2023).

**TABLE 1:** Classification of diuretics based on mechanism of action: validated and non-validated therapeutic indications^a^.

Classes of diuretics	Validated indications	Non-validated indications
molecule(s)		
**Osmotic diuretics**	Intracranial hypertension	Oliguric renal failure
Mannitol		
**Loop diuretics**	Salt and water overload, Acute oliguric renal failure, Electrolyte disorders	Post-hemorrhagic hydrocephalus, Bronchopulmonary dysplasia, Chronic lung disease, Hyaline membrane disease, Prophylactic use during blood transfusion
Furosemide, Bumetanide		
**Thiazide diuretics**	Salt and water overload, Proximal renal tubular acidosis, Nephrogenic diabetes insipidus	Nephrocalcinosis, Bronchopulmonary dysplasia, Chronic lung disease
Chlorothiazide, Hydrochlorothiazide		
**Carbonic anhydrase inhibitors**	Metabolic alkalosis, Hypochloremia	Post-hemorrhagic hydrocephalus
Acetazolamide		
**K^+^ sparing**	Antikaliuretic, Nephrogenic diabetes insipidus	Chronic lung disease, Bronchopulmonary dysplasia
Spironolactone, Amiloride		
**Xanthines**	Hypoxemic renal failure	Hyaline membrane disease, Respiratory distress syndrome, Transient tachypnoea
Caffeine, Theophylline, Aminophylline		
**Filtration diuretics**	Low blood pressure, Oligo-anuria	Fluid retention
Dopamine		

^a^ Modified from reference 1.

Based on the literature, several side effects of diuretic agents are reported. Furosemide is among the 20 most commonly used drugs in neonatology (Gouyon et al., 2019). This loop diuretic is often administered at doses higher than recommended (Manfredini et al., 2020), and it is one of the most frequently prescribed off-label drugs in the neonatal intensive care unit (NICU) (Krzyżaniak et al., 2016). Neonatal exposure to furosemide is associated to electrolyte disorders (Sridharan et al., 2022) and nephrocalcinosis (Gimpel et al., 2010). Furthermore, several studies report that exposure to high doses of furosemide is a risk factor for ototoxicity, even if the exact dose causing hearing loss has not been determined yet for newborn infants (Wang et al., 2018; Manfredini et al., 2020; Khan et al., 2023). The duration of treatment is an additional risk factor for the occurrence of side effects. Indeed, according to an Italian national survey on the use of furosemide in NICUs, close monitoring of side effects is necessary if the exposure to furosemide exceeds 14 days in extremely preterm infants (Manfredini et al., 2020). Many studies report the increasing use of diuretics in NICUs, as well as a high variability in practices. These studies are characterized by small sample sizes, declarative data, retrospective (from records), and non-exhaustive information on indications and cumulative doses (Hagadorn et al., 2011; Slaughter et al., 2013; Laughon et al., 2015; Krzyżaniak et al., 2016; Greenberg et al., 2019; Greenberg et al., 2020; Tan et al., 2020; Bamat et al., 2021). To our knowledge, there is a lack of epidemiologic data on diuretic utilization patterns and on detailed exposure rates to diuretics in neonates.

The main objective of our study was to measure in a large cohort of newborn infants, the rate of exposure to diuretics according to the prescription data of 40 level 3 French NICUs using the same computerized prescription order entry (CPOE) system. The secondary objectives were to analyze the evolution of this exposure over time, describe the indications, prescription modalities, and variations between different hospital centers.

## 2 Materials and methods

### 2.1 Study type and data sources

This was an observational multicenter cohort study with the participation of 40 level 3 French NICUs using the same CPOE software Logipren^®^. This software allows for the prescription of medications based on indication, gestational age, postnatal age, birth weight, and body weight on the day of prescription (Gouyon et al., 2019). Posology and indications are based on a regularly updated “reference thesaurus” developed from national (marketing authorizations, National Agency for the Safety of Medicines and Health Products, French Society of Neonatology) and international recommendations. All entered prescriptions are prospectively recorded and stored at each hospital site. Monthly, all prescription data is extracted, pseudonymized, and centralized in a database hosted by a data center certified for health data (TESIS e-health, La Réunion).

### 2.2 Study population and study period

The study population consisted of all newborn infants hospitalized in 40 level 3 French NICUs using the software Logipren^®^. The eligibility criteria were: hospitalization in a level 3 NICU, admission between 01/01/2017 and 12/31/2021, neonates with a postnatal age between 24 and 44 weeks of gestation (WG). The inclusion period was from 1 January 2017, to 31 December 2021.

### 2.3 Outcomes

The main outcome of interest was the rate of exposure to diuretics. Secondary outcomes were: evolution of exposure over time, indications, prescription modalities, variations between different hospital centers. Exposure to diuretics was defined as the prescription, at least once, of a medication among the following: acetazolamide, aminophylline, bumetanide, caffeine, dopamine, furosemide, hydrochlorothiazide, mannitol, spironolactone and theophylline, with a diuretic purpose. The variable “indication” was categorized as “validated” or “non-validated” if effectiveness was proven in the literature or not, respectively (Guignard and Iacobelli, 2021). Duration of exposure was classified by total days of exposure to at least one diuretic. Cumulative dose was calculated as the sum of all doses (mg/kg) received by the patient during the hospital stay.

### 2.4 Statistical analysis

Qualitative and ordinal variables were described in terms of frequency and percentage. Quantitative variables were described in terms of mean and standard deviation. The 95% confidence intervals (95% CI) were calculated for the main outcome. For comparisons between two independent groups, the following tests were performed at a significance level set at 5%: The Chi-squared test for qualitative variables, the Student’s *t*-test for quantitative variables, and the Cochran-Armitage test for the evolution of exposure over time. Statistical analysis was conducted using SAS^®^ software (SAS Institute, version 9.4, North Carolina, United States).

### 2.5 Ethics statement

This study was conducted in accordance with the French law applicable to human research (Jardé Act). The specific approval of an ethics committee is not required for this non-interventional study based on anonymized data of authorized collections (declaration number CNIL: DE-2015-099, DE-2017-410), and written parental consent is not needed.

## 3 Results

### 3.1 Characteristics of the study population and rate of exposure to diuretics

A total of 86,032 patients met our inclusion criteria and constituted our study population (Table 2). This population was predominantly male (54.5%) with an average birth weight of 2.45 kg and an average gestational age of 35.4 WG. The hospital stay of patients had an average duration of 15.8 days with an in-hospital mortality rate of 2.9%. In total, 7,313 newborns (8.5%) had at least one prescription of diuretics during hospitalization. It is worth noting a decrease in the exposure rate as gestational age increased. Indeed, 52.6% of extremely preterm infants (24-27 WG) were exposed to diuretics compared to 3.7% of full-term infants (≥37 WG).

**TABLE 2:** Characteristics of the study population and exposure rates to diuretics.

	Study population *n* = 86,032	Gestational age [weeks of gestation]			
		[24–27] *n* = 5,775	[28–31] *n* = 10,426	[32–36] *n* = 28,855	≥37 *n* = 40,976
Male, n (%)	46,920 (54.5)	3,045 (52.7)	5,599 (53.7)	15,372 (53.3)	22,904 (55.9)
Birth weight (kg), mean (±SD)	2.45 (0.95)	0.81 (0.19)	1.31 (0.32)	2.15 (0.51)	3.19 (0.59)
Length of stay (days), mean (±SD)	15.8 (20.8)	48.5 (40.4)	34.7 (24.5)	15.0 (13.6)	6.8 (8.8)
Mortality, n (%)	2,509 (2.9)	1,139 (19.7)	370 (3.5)	386 (1.3)	614 (1.5)
Exposure to diuretics, n (%)	7,313 (8.5)	3,039 (52.6)	1,721 (16.5)	1,022 (3.5)	1,531 (3.7)
[CI 95%]	[8.3; 8.7]	[51.3; 53.9]	[15.8; 17.2]	[3.3; 3.8]	[3.6; 3.9]

CI, confidence interval; SD, standard deviation.

### 3.2 Characteristics of patients exposed to diuretics

The exposure rate of the study population varied from 8.2% to 9.4% across the years. There was no significant difference in exposure rates over time (*p* < 0.19) (data not shown).

Among the exposed patients, 58.3% were male (Table 3). Mean birth weight was 1.56 kg and mean gestational age was 30.4 WG. The hospital stay for these patients was on average 46.4 days, with an in-hospital mortality rate of 16.4%. There were key differences between exposed and not-exposed infants, regarding sex, gestational age, birth weight, length of hospitalization and mortality.

**TABLE 3:** Characteristics of patients exposed to diuretics vs. non-exposed patients.

	Patients exposed to diuretics		*p*-value*
	No *n* = 78,719	Yes *n* = 7,313	
Male, n (%)	42,654 (54.2)	4,267 (58.3)	<0.0001
Birth weight (kg), mean (±SD)	2.54 (0.90)	1.56 (1.01)	<0.0001
Gestational age (weeks), mean (±SD)	35.8 (3.9)	30.4 (5.3)	<0.0001
Length of stay (days), mean (±SD)	12.9 (15.7)	46.4 (37.7)	<0.0001
Mortality, n (%)	1,308 (1.7)	1,201 (16.4)	<0.0001

SD, standard deviation.

### 3.3 Diuretic prescription modalities

In total, 10 different international non-proprietary names (INNs) were prescribed during the study period (Table 4). The most prescribed INNs were furosemide and spironolactone administered in 76.8% and 34.7% of patients exposed to diuretics, respectively. The most commonly used administration route was injectable intravenous. More than half (60.7%) of exposed patients received their first prescription after the first week of life, and the average exposure duration was 11.2 days. Nearly a third (31.8%) received two or more diuretics during hospitalization. Most indications were non-validated, mainly for cases of “fluid retention,” “BPD,” and “post-transfusion.” Side effects were reported for 0.7% of diuretic-exposed patients, mostly involving hydroelectrolyte and/or acid-base balance disorders. Other reported adverse effects were acute renal failure (2 patients), nephrocalcinosis (2 patients) and hypotension (1 patient).

**TABLE 4:** Description of diuretics prescription.

	Patients exposed to diuretics *n* = 7,313	Gestational age [weeks of gestation]			
		[24–27] *n* = 3,039	[28–31] *n* = 1,721	[32–36] *n* = 1,022	≥37 *n* = 1,531
Diuretics INN, n (%)					
Furosemide	5,614 (76.8)	2,286 (75.2)	1,136 (66.0)	821 (80.3)	1,371 (89.5)
Spironolactone	2,536 (34.7)	1,349 (44.4)	820 (47.6)	208 (20.4)	159 (10.4)
Dopamine	1,488 (20.3)	902 (29.7)	251 (14.6)	154 (15.1)	181 (11.8)
Bumetanide	199 (2.7)	78 (2.6)	28 (1.6)	33 (3.2)	60 (3.9)
Hydrochlorothiazide	101 (1.4)	64 (2.1)	32 (1.9)	3 (0.3)	2 (0.1)
Aminophylline	68 (0.9)	11 (0.4)	5 (0.3)	13 (1.3)	39 (2.5)
Acetazolamide	28 (0.4)	7 (0.2)	5 (0.3)	8 (0.8)	8 (0.5)
Mannitol	9 (0.1)	1 (0.0)	0 (0.0)	2 (0.2)	6 (0.4)
Caffeine	3 (0.0)	0 (0.0)	0 (0.0)	0 (0.0)	3 (0.2)
Theophylline	1 (0.0)	0 (0.0)	0 (0.0)	1 (0.1)	0 (0.0)
Route of administration, n (%)					
Intravenous	5,672 (77.6)	2,457 (80.8)	1,079 (62.7)	804 (78.7)	1,332 (87.0)
Enteral	3,350 (45.8)	1,551 (51.0)	1,009 (58.6)	362 (35.4)	428 (28.0)
Inhaled	14 (0.2)	12 (0.4)	2 (0.1)	0 (0.0)	0 (0.0)
Post-natal age at first diuretic prescription (days), n (%)					
Post-natal age at first diuretic prescription (days), mean (±SD)	16.7 (18.3)	20.5 (21.7)	20.7 (16.0)	12.1 (14.0)	7.6 (10.3)
[D1; D2]	1,313 (18.0)	383 (12.6)	216 (12.6)	269 (26.3)	445 (29.1)
[D3; D7]	1,558 (21.3)	586 (19.3)	187 (10.9)	241 (23.6)	544 (35.5)
≥ D8	4,442 (60.7)	2,070 (68.1)	1,318 (76.6)	512 (50.1)	542 (35.4)
Duration of exposure to diuretics (days), mean (±SD)	11.2 (16.4)	15.0 (19.9)	10.6 (13.7)	8.4 (14.3)	6.3 (9.4)
Indications, n (%)					
Non-validated	5,644 (77.2)	2,489 (81.9)	1,457 (84.7)	705 (69.0)	993 (64.9)
Validated	3,234 (44.2)	1,418 (46.7)	521 (30.3)	497 (48.6)	798 (52.1)
Type of non-validated indications, n (%)					
Fluid retention	5,267 (72.0)	2,280 (75.0)	1,347 (78.3)	669 (65.5)	971 (63.4)
Bronchopulmonary dysplasia	520 (7.1)	346 (11.4)	151 (8.8)	19 (1.9)	4 (0.3)
Post-transfusion	302 (4.1)	188 (6.2)	60 (3.5)	28 (2.7)	26 (1.7)
Other	25 (0.3)	9 (0.3)	6 (0.3)	5 (0.5)	5 (0.3)
Post-hemorrhagic hydrocephalus	5 (0.1)	1 (0.0)	2 (0.1)	2 (0.2)	0 (0.0)
Type of validated indications, n (%)					
Oligo-anuria/Low blood pressure^a^	1,488 (20.3)	902 (29.7)	251 (14.6)	154 (15.1)	181 (11.8)
Salt and water overload	1,393 (19.0)	484 (15.9)	183 (10.6)	252 (24.7)	474 (31.0)
Acute oliguric renal failure	488 (6.7)	218 (7.2)	71 (4.1)	75 (7.3)	124 (8.1)
Salt and water overload and/or Antikaliuretic	219 (3.0)	81 (2.7)	41 (2.4)	45 (4.4)	52 (3.4)
Hyperaldosteronism	77 (1.1)	35 (1.2)	30 (1.7)	11 (1.1)	1 (0.1)
Hypoxemic renal failure	70 (1.0)	11 (0.4)	5 (0.3)	13 (1.3)	41 (2.7)
Electrolyte disorders	35 (0.5)	7 (0.2)	5 (0.3)	13 (1.3)	10 (0.7)
Metabolic alkalosis	22 (0.3)	6 (0.2)	3 (0.2)	6 (0.6)	7 (0.5)
Intracranial hypertension	9 (0.1)	1 (0.0)	0 (0.0)	2 (0.2)	6 (0.4)
Nephrogenic diabetes insipidus	1 (0.0)	0 (0.0)	0 (0.0)	0 (0.0)	1 (0.1)
Side effects, n (%)	48 (0.7)	27 (0.9)	10 (0.6)	6 (0.6)	5 (0.3)

D, day of life; INN, international non-proprietary names; SD, standard deviation.

^a^ Oligo-anuria in a context of hypotension or glomerular hypoperfusion (validated indication for dopamine).

Prescription of furosemide was analyzed more specifically (Table 5). This drug was prescribed to 63.7% of exposed patients after the first week of life. The most used administration route was injectable intravenous, and the total administered dose of furosemide averaged 10.7 mg/kg. It is noteworthy that 43.6% of these patients received a cumulative furosemide dose exceeding 4 mg/kg, and 23.5% had a cumulative dose exceeding 10 mg/kg. The average duration of furosemide treatment was 6.2 days, and 9.7% of these patients had a treatment duration exceeding 14 days. The indication for furosemide was non-validated in 81.9% of exposed patients, mainly for fluid retention. Side effects were observed in 0.4% of exposed patients, primarily hydroelectrolyte and/or acid-base balance disorders.

**TABLE 5:** Description of furosemide exposure.

	Patients exposed to furosemide *n* = 5,614	Gestational age [weeks of gestation]			
		[24–27] *n* = 2,286	[28–31] *n* = 1,136	[32–36] *n* = 821	≥37 *n* = 1,371
Route of administration, n (%)					
Intravenous	4,897 (87.2)	2,088 (91.3)	925 (81.4)	690 (84.0)	1,194 (87.1)
Enteral	1,494 (26.6)	538 (23.5)	356 (31.3)	232 (28.3)	368 (26.8)
Inhaled	14 (0.2)	12 (0.5)	2 (0.2)	0 (0.0)	0 (0.0)
Post-natal age at first furosemide prescription (days), mean (±SD)	16.5 (17.8)	20.9 (20.5)	20.5 (17.0)	12.4 (14.4)	8.2 (10.4)
Post-natal age at first furosemide prescription (days), n (%)					
[D1; D2]	714 (12.7)	116 (5.1)	100 (8.8)	183 (22.3)	315 (23.0)
[D3; D7]	1,324 (23.6)	417 (18.2)	153 (13.5)	220 (26.8)	534 (38.9)
≥ D8	3,576 (63.7)	1,753 (76.7)	883 (77.7)	418 (50.9)	522 (38.1)
Total administered dose of furosemide (mg/kg), mean (±SD)	10.7 (23.9)	8.8 (20.4)	8.5 (18.9)	15.6 (35.4)	12.8 (23.7)
Total administered dose of furosemide (mg/kg), n (%)					
Not provided	5 (0.1)	3 (0.1)	2 (0.2)	0 (0.0)	0 (0.0)
[0; 4]	3,160 (56.3)	1,298 (56.8)	746 (65.7)	436 (53.1)	680 (49.6)
]4; 10]	1,129 (20.1)	496 (21.7)	192 (16.9)	143 (17.4)	298 (21.7)
>10	1,320 (23.5)	489 (21.4)	196 (17.3)	242 (29.5)	393 (28.7)
Duration of exposure to furosemide (days), n (%)					
Duration of exposure to furosemide (days), mean (±SD)	6.2 (9.5)	6.0 (8.2)	5.2 (7.9)	8.1 (14.1)	6.1 (9.0)
[1; 14]	5,068 (90.3)	2,086 (91.3)	1,049 (92.3)	703 (85.6)	1,230 (89.7)
>14	546 (9.7)	200 (8.7)	87 (7.7)	118 (14.4)	141 (10.3)
Indications, n (%)					
Non-validated	4,597 (81.9)	2,030 (88.8)	1,016 (89.4)	610 (74.3)	941 (68.6)
Validated	1721 (30.7)	606 (26.5)	231 (20.3)	314 (38.2)	570 (41.6)
Type of non-validated indications, n (%)					
Fluid retention	4,366 (77.8)	1,912 (83.6)	951 (83.7)	583 (71.0)	920 (67.1)
Post-transfusion	302 (5.4)	188 (8.2)	60 (5.3)	28 (3.4)	26 (1.9)
Bronchopulmonary dysplasia	110 (2.0)	70 (3.1)	37 (3.3)	3 (0.4)	0 (0.0)
Other	25 (0.4)	9 (0.4)	6 (0.5)	5 (0.6)	5 (0.4)
Type of validated indications, n (%)					
Salt and water overload	1,270 (22.6)	423 (18.5)	164 (14.4)	235 (28.6)	448 (32.7)
Acute oliguric renal failure	463 (8.2)	207 (9.1)	67 (5.9)	72 (8.8)	117 (8.5)
Electrolyte disorders	35 (0.6)	7 (0.3)	5 (0.4)	13 (1.6)	10 (0.7)
Side effects, n (%)	24 (0.4)	12 (0.5)	2 (0.2)	5 (0.6)	5 (0.4)

D, day of life; SD, standard deviation.

### 3.4 Exposure rates across NICUs

**FIGURE 1:**
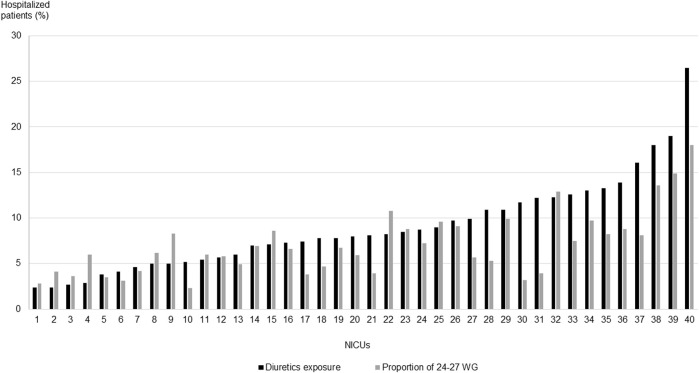
Diuretic exposure rates and proportion of 24–27 weeks of gestation patients cared for in each NICU.

There was variability in the exposure rates to diuretics among different hospital centers (shown in Figure 1). The median exposure to diuretics per NICU was 8.1%, ranging from 2.4% to 26.5%. We also observed a correlation (*r* = 0.77) between the exposure rate to diuretics and the proportion of extremely preterm infants cared for in each NICU.

## 4 Discussion

Our study described diuretic prescription practices over a 5-year period in a cohort of newborn infants hospitalized in 40 French NICUs. The overall exposure rate was 8.5% varying from 3.7% in full-term infants to 52.6% in extremely preterm infants. The main characteristics of exposed children were a lower gestational age and birth weight compared to the non-exposed group. This observation can be explained by the increased morbidity and the increased rate of critical illness in preterm and VLBW infants compared to full-term ones. A retrospective cohort study conducted in 2015 in the United States using data from the Pediatrix Medical Group reported a diuretic exposure rate of 37% (Laughon et al., 2015). This study focused on infants born at <32 WG and <1.500 g birth weight, hospitalized in 333 NICUs between 1997 and 2011. Their findings slightly differed from the exposure rate found in our study, which was 29% in neonates of the same gestational age. This difference may be explained by the fact that the US study was carried out several years before the present one, and over decades, changes in neonatal intensive care prescription practices have been observed (Stark et al., 2022). Moreover, the exposure rate reported by the Pediatrix Medical Group was potentially underestimated, as it was not based on the analysis of actual prescription data, but rather on information extracted from physicians’ entries in an electronic medical record (Spitzer et al., 2015). Our study showed that over time, diuretic exposure tended to remain stable over a 5-year period. A similar finding was reported in the study by Laughon et al. (2015), where the exposure rate did not significantly differ from 2005 (39%) to 2011 (36%).

The results of our study confirm that diuretics remain among the most frequently prescribed drugs in NICUs (Gouyon et al., 2019), despite the lack of new evidence of their effectiveness. In our cohort, the most frequently prescribed diuretic was furosemide, followed by spironolactone. This finding is consistent with results from previous research (Pacifici, 2013; Girardi et al., 2015; Laughon et al., 2015; Manfredini et al., 2020). Our cohort is the first to describe the exposure period and indications for each diuretic molecule used. In our study, diuretics were mainly prescribed after the first week of life and, in most cases, with a “non-validated” indication. It is worth noting that the main non-validated indications were “fluid retention” and “BPD.” This may be explained by the empirical medical observation of “fluid retention” associated with BPD, often reported in the literature (Greenberg et al., 2020), and the established idea that the use of diuretics could consequently improve respiratory function in this condition (Stewart and Brion, 2011; Stewart et al, 2011; Slaughter et al. 2013; Manfredini et al., 2020). Indeed, although literature data (Stewart and Brion, 2011; Stewart et al, 2011) do not encourage the use of diuretics in this pathology, these drugs are widely used to improve the pulmonary function of preterm infants with BPD (Stewart and Brion, 2011; Manfredini et al., 2020; Tan et al., 2020). We noted a significant variability in the use of diuretics among different hospital centers, consistent with results observed in previous studies reporting a variation of 0%–75% in exposure rates for preterm infants with gestational age <32 WG and birth weight <1,500 g (Laughon et al., 2015). However, we found a positive correlation between the proportion of extremely preterm infants cared for in each facility and the diuretic exposure rate. We can suggest that the care of the most vulnerable patients may influence the frequency of diuretic use.

The cumulative dose of furosemide and the duration of exposure can be risk factors for adverse events. Previous research in small, single-center observational studies, has reported that patients experienced electrolyte disturbances after exposure to furosemide at a cumulative dose >4 mg/kg (Sridharan et al., 2022), and newborns had a 48 times higher risk of developing nephrocalcinosis after exposure to furosemide at a cumulative dose >10 mg/kg (Gimpel et al., 2010). We noticed that 43% of exposed patients in our study received a cumulative dose >4 mg/kg, and nearly a quarter received a dose >10 mg/kg. In our study, the rate of children with a furosemide treatment duration >14 days was 9.7%, and unfortunately, we do not have information on the follow-up of our cohort. There is a low rate of side effects in our cohort compared to other studies (Gimpel et al., 2010; Sridharan et al., 2022). It is likely that reporting of side effects based on the Logipren^®^ database is underestimated, as it can only be done at the time of drug discontinuation, while the deleterious consequences of diuretic exposure (especially ototoxicity and nephrocalcinosis) can manifest several days or weeks post-treatment. This represents a limitation of our research. Another limitation is the lack of information on the main morbidities associated with preterm birth, and more generally on the prevalence in the studied population of pathologies that may benefit from diuretic treatment (renal failure, hypertension, etc.). Finally, our data did not allow us to differentiate the indication “established” or “evolving” BPD and this information would have been interesting for the interpretation of our results.

Our study has several strengths. The main one was the use of a large set of recent data obtained from 40 Level 3 NICUs, representing nearly 60% of existing Level 3 NICUs in France (DREES, 2023). Additionally, the data were collected prospectively on a real prescription database. We also note that the number of missing data is close to zero in our database.

## 5 Conclusion

This study is the first national cohort describing diuretic prescription practices in neonates. Our results allow us to conclude that diuretics are still widely used in NICUs and their prescription is often off-label, despite the lack of evidence of their efficacy, especially for the treatment of BPD. There is considerable variability in prescription practices among different French hospital centers. Further studies are needed to understand the short- and long-term effects of diuretic treatment and to establish recommendations on dosage, indication, and duration of treatment.

## Data Availability

The raw data supporting the conclusion of this article will be made available by the authors, without undue reservation.
